# Chyle leakage after axillary node sampling in a patient with breast cancer: a case report

**DOI:** 10.1186/s40792-020-00885-y

**Published:** 2020-06-01

**Authors:** Norio Kohno, Takeo Kimoto, Akiko Okamoto, Hirokazu Tanino

**Affiliations:** 1 Department of Breast Surgery, Kobe Kaisei Hospital, 3-11-15 Shinohara-Kitamachi, Nada-ku, Kobe, Hyogo 657-0068 Japan; 2 Department of Breast Surgery, Kobe University Hospital, 7-5-2 Kusunoki-cho, Chuo-ku, Kobe, Hyogo 650-0017 Japan

**Keywords:** Chyle leakage, Breast cancer, Axillary node sampling, Surgical treatment, Case report

## Abstract

**Background:**

Chyle leakage is a well-known complication after thoracic surgery, such as esophagectomy, cardiac surgery, mediastinal lymph node dissection, and neck surgery. However, chyle leakage is a rare complication after dissections of the lateral or subclavian axillary nodes for breast surgery. It is particularly unusual for chyle leakage to occur after minimally invasive dissection of the axillary nodes. Most cases of chyle leakage subside with conservative management, but some cases require surgery.

**Case report:**

An 80-year-old woman had invasive lobular cancer of the left breast (cT1 [1.7 cm], cN0, M0) for which she underwent breast-conservative surgery and biopsy of an axillary sentinel lymph node. Because two of the three sentinel lymph nodes tested positive for cancer, seven lateral axillary lymph nodes (level I) were subsequently removed for the additional sampling. On postoperative day 11, the patient visited our outpatient clinic because of swelling in her left axillary region and breast. Centesis of the axilla yielded 670 mL of milky fluid, which suggested chyle leakage. We commenced the conservative management at first; however, the persistent leakage made us perform the surgical management. The operation was not only ligating the opening of the chyle duct but needed total mastectomy because the postoperative pathology report showed invasive lobular carcinoma; the nipple and the caudal surgical margin of the lumpectomy were positive for cancer. The patient agreed to our recommendation of total mastectomy and surgical management of the chyle leakage. Ligation of the opening completely resolved the chylous discharge.

**Conclusion:**

We here report a case of large-volume leakage of chyle after sampling dissection of the lateral axillary lymph nodes for left breast cancer; the leakage persisted despite the standard conservative therapy but was resolved after surgical treatment. Chyle leakage can occur even after minimally invasive dissection of the axillary nodes.

## Background

Surgical procedures for breast cancer are tending to become less invasive. For example, total dissection of the axillary nodes—which was previously routine in patients with breast cancer—may not contribute to survival [1]. However, metastasis to axillary lymph nodes remains a valuable indicator of the aggressiveness of a patient’s disease, and the number of metastatic nodes is an important indicator of the required intensity of the postoperative treatment regimen. Consequently, the axillary lymph node sampling and removal remains to be an important indicator on deciding the treatment policies for some patients.

Most chyle leaks after axillary node dissection are associated with the dissection of level II axillary nodes (those lying underneath the pectoralis minor muscle) or higher. Here, we report a case of high-volume chyle leakage from the left axilla after only sampling of the lateral axillary lymph nodes additionally performed due to cancer-positive sentinel nodes. The leakage persisted despite standard conservative therapy but resolved after surgical treatment.

## Case history

An 80-year-old woman was diagnosed with invasive lobular cancer of the left breast through the ultrasound-guided core needle biopsy. Her preoperative clinical diagnosis was stage I: cT1 (1.7 cm), cN0, M0, and unilateral, unifocal lobular carcinoma was confirmed through the ultrasound and the magnetic resonance mammography examinations. On immunochemistry, the tumor cells were ER 99%, PgR 98%, HER2 negative, and Ki67 index 5.9%. The patient underwent breast-conserving surgery to remove the 1.7-cm tumor in the outer upper region of her left breast, and her axillary sentinel lymph nodes were biopsied by using the blue dye only. Because two of three sentinel lymph nodes tested positive for cancer according to the one-step nucleic acid amplification assay [2], a total of seven nodes were removed during sampling dissection of the lateral axillary lymph nodes (level I). Postoperatively, the fluid in the axillary cavity drain was 178 mL per day on postoperative day 1 (POD), bloody in color as usual, then the volume of drainage started to decrease, though, and the fluid seemed to be slightly milky in color from POD 3. The drain was removed POD 6 with 60 mL in volume per day. The patient was discharged on POD 7, as is typical our operative course.

**Fig. 1:**
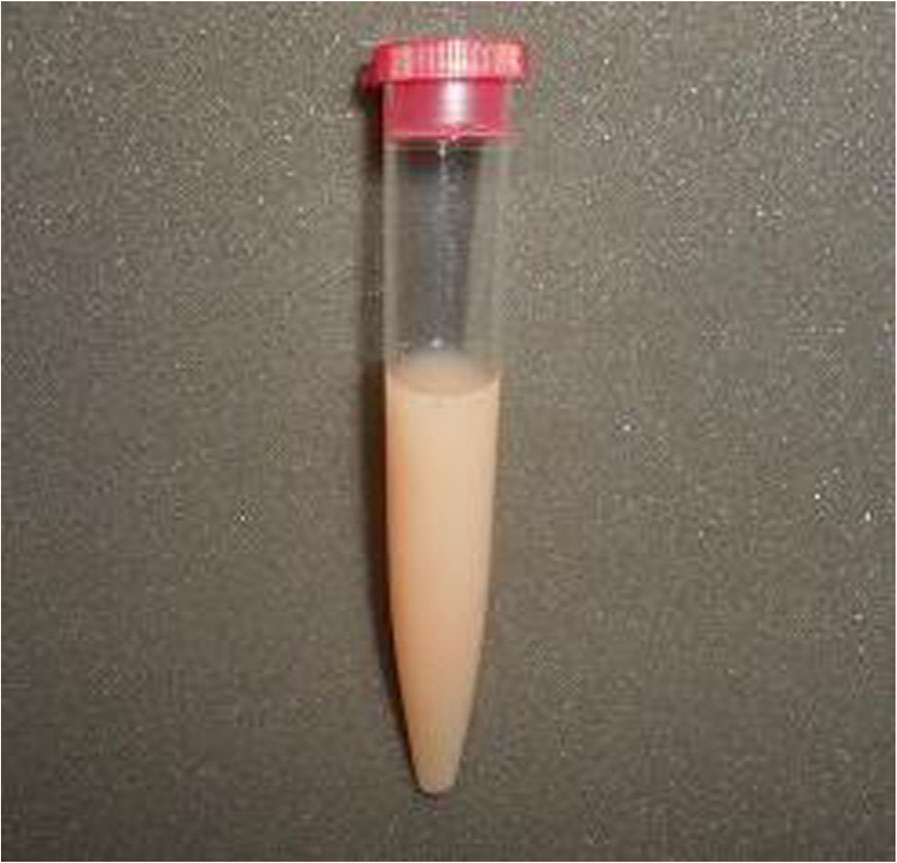
Appearance of the milky aspirate

**Fig. 2:**
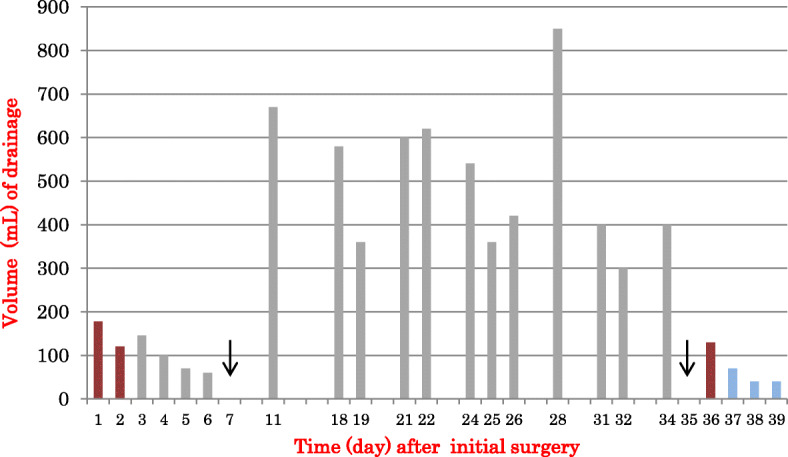
Volume of the bloody drainage fluid (red bars) and milky drainage fluid (gray bars) after the initial surgery. The fluid was slightly milky in color from POD 3. The drain was removed POD 6 with 60 mL per day. The patient was discharged on postoperative day (POD) 7(black arrow). Total mastectomy was done on POD 35 (black arrow). After POD 36, the blue bars indicate the volume of the discharge from the drain; this fluid was serous rather than milky in color

Four days after the discharge, on POD 11, the patient visited our outpatient clinic because of swelling in her left axillary region and breast. Centesis of the axilla yielded 670 mL of milky fluid. The results of biochemical comparisons between the aspirate and the serum are presented below, together with the normal serum level ranges: total cholesterol (normal serum level, 120–219 mg/dL), 36 vs. 156 mg/dL; HDL cholesterol (41–90 mg/dL), 6 vs. 50 mg/dL; triglycerides (50–149 mg/dL), 513 vs. 86 mg/dL; and LDL cholesterol (70–139 mg/dL), 1 vs. 94 mg/dL. Both the milky appearance of the aspirate and its high triglyceride level supported the diagnosis of chyle leakage. We commenced the conservative management at first, comprising a thoracic compression band and a low-fat diet. Despite these procedures, high-volume chyle discharge had not subsided, which, consequently, made us perform the surgical management. Considering the final pathological result, we completed left total mastectomy, which showed that she had invasive lobular carcinoma and that 7 (including the sentinel nodes) of 10 level I lymph nodes were metastatic. In addition, the nipple and the caudal margin of the surgical specimens were positive for cancer. The histopathological diagnosis was pT2pN2a (7/10) M0. The patient agreed to our recommendation of total mastectomy and surgical management of the chyle leakage (Figs. 1 and 2).

**Fig. 3:**
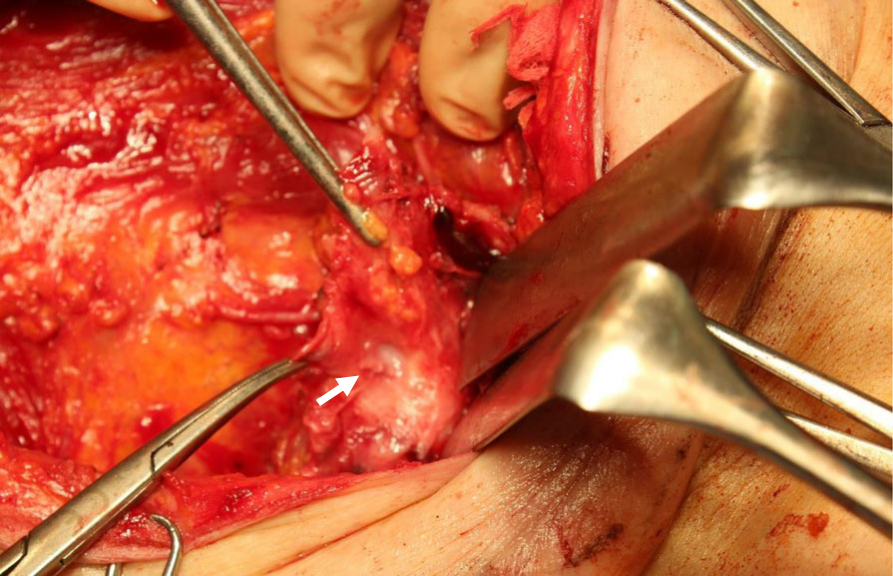
Operative findings. The white arrow indicates the opening responsible for the chyle leakage; milky fluid flows from the nicked duct

**Fig. 4:**
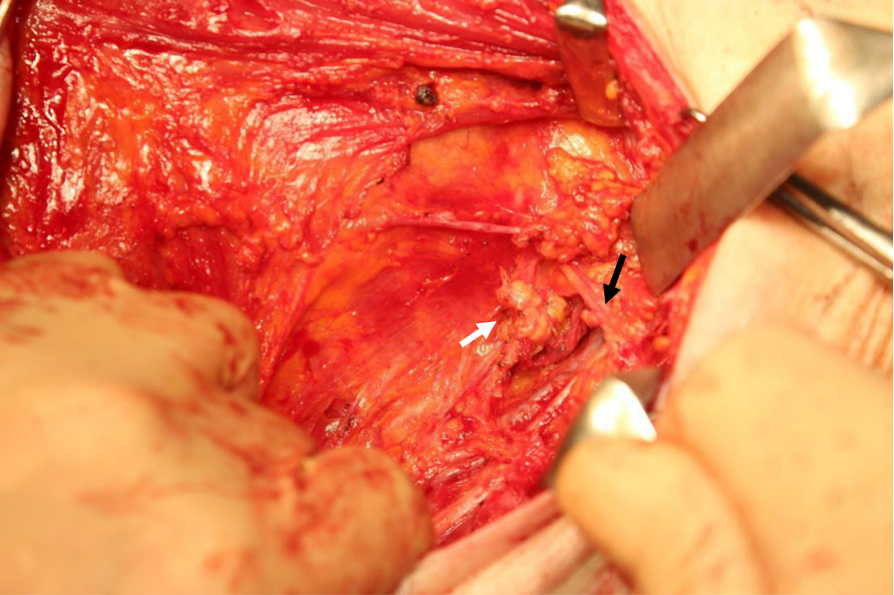
Operative findings. The white arrow indicates ligation of the opening; the black arrow indicates the 2nd intercostobrachialnerves

The operation was performed on POD 35. Because the patient had eaten ice cream 3 h before surgery, we were able to locate the opening causing the chylous leakage. This opening was approximately 3 mm in diameter and located below the 2nd intercostobrachialnerves (Figs. 3 and 4). Ligation of the opening completely resolved the chylous discharge. We removed the axillary drain 5 days after the total mastectomy. Invasive lobular carcinoma remained only in the nipple and caudal portions, close to the post-lumpectomy scar; the cancerous area measured 2 mm in depth and 1 mm in diameter according to the histopathological examination of the total segmented specimens.

Postoperative therapy included the use of an aromatase inhibitor and radiotherapy of the chest wall and axillary, infraclavicular, supraclavicular, and parasternal lymph nodes.

## Discussion

Chyle leakage due to iatrogenic injury of the thoracic duct (TD) is typically associated with neck surgery. Whereas the incidence of chyle leakage after neck surgery is 2–8% [3, 4], this complication is rare after axillary node dissection (0.36–0.84%) [5].

The TD is the largest lymphatic vessel in the human body and daily transports approximately 3 L of lymph back to the systemic circulation [6]. The TD begins in the abdomen, ascending along the thoracic spine until reaching the base of the neck. Anatomically, the TD demonstrates many variations. In its terminal course, the TD exists as a single duct in 87.5% of cases, whereas 12.5% have multiple (double, 8.5%; triple, 1.8%; and quadruple, 2.2%) terminations. Of the TDs reported in the literature, 90% have at least one termination into the internal jugular vein, subclavian vein, or venous angle. Other additional variations in the TD terminus characteristically occur within 1–2 cm of the junction of the left subclavian vein and internal jugular vein. In 10% of humans, the TD terminates in the suprascapular, brachiocephalic, vertebral, or transverse cervical veins or in the cervical lymph chain. Right-sided TD terminations occur in only 1–6% of humans [7]. These variations are the reason for the rare incidence of chyle leakage after axillary lymph node dissection and why it rarely occurs after surgery on the right side of the body. Many authors suggest that chyle leakage is typically due to injury of the subclavian duct or its tributaries, which lie near the level II lymph nodes, and the most reported chyle leaks are associated with level II axillary node dissection or higher [5]. However, one reported case of chyle leakage occurred after biopsy of the four left axillary sentinel lymph nodes in a breast cancer patient with superior vena caval thrombosis [8]. In the case we present here, the patient had no past medical procedures, such as neck or thoracic surgery that might change the path of her TD. We consider that the chyle leakage was not related to cancer involvement of axillary nodes because TD and axillary lymphatics do not communicate anatomically with each other. Rather, we consider that a rare anatomical variation of TD routes may have led to the chyle leakage in our patient.

The minimally invasive axillary lymph node dissection that we performed in our patient is similar to the level I sampling. The optimal management of the axilla remains unclear. In the case presented, complete axillary lymph node dissection (ALND) was not performed due to the patient’s age and to avoid complications with ALND, instead, the postoperative radiotherapy of the regional lymph nodes might have been a better option [9, 10].

The diagnosis of chyle leakage depends on the milky appearance of the drainage fluid, and high levels of triglycerides (> 100 mg/dL higher than serum levels) in the fluid provide supporting evidence [11]. Most cases of chyle leakage resolve after conservative treatment, including low-fat diet, parenteral nutrition for dietary restriction, and pressure on the chest and axillary cavity; some cases of chyle leakage resolve spontaneously [12]. Octreotide, a somatostatin analog, is a candidate agent for the treatment of chyle leakage, but the optimal dosage and route of administration have not yet been confirmed [13].

There is no consensus regarding the indication and timing of surgical treatment for chyle leakage. Our patient consequently required the repair of the lymph vessel for ceasing the intractable chyle leakage combined with performing total mastectomy due to tumor-positive surgical margins. When, as in our case, high-volume chyle leakage persists for more than 14 days despite conservative therapy, surgical treatment may be warranted.

## Conclusion

The incidence of chyle leakage after breast cancer surgery likely will decrease, given the results of the ACOSOG Z0011 trial is duly evaluated, which showed that the standard surgical treatment of decreased axillary lymph node dissection is appropriate for breast cancer in terms of survival [9]. However, as our case demonstrates, chyle leakage can still occur after minimally invasive procedures for breast cancer, including biopsy of axillary sentinel lymph nodes [8]. We propose surgical intervention when chyle leakage persists despite conservative treatment.

## Abbreviations

ALND: Axillar lymph node dissection
ER: Estrogen receptor
HER2: Human epidermal growth factor receptor 2
HDL cholesterol: High-density lipoprotein cholesterol
LDL cholesterol: Low-density lipoprotein cholesterol
PgR: Progesterone receptor
POD: Postoperative day
TD: Thoracic duct
